# Do prescription stimulants increase the risk of adverse cardiovascular events?: A systematic review

**DOI:** 10.1186/1471-2261-12-41

**Published:** 2012-06-09

**Authors:** Arthur N Westover, Ethan A Halm

**Affiliations:** 1 Department of Psychiatry, UT Southwestern Medical Center, Dallas, TX, USA; 2 Division of Outcomes and Health Services Research, Department of Clinical Sciences, UT Southwestern Medical Center, Dallas, TX, USA; 3 Division of General Internal Medicine, Department of Internal Medicine, UT Southwestern Medical Center, Dallas, TX, USA

## Abstract

**Background:**

There is increasing concern that prescription stimulants may be associated with adverse cardiovascular events such as stroke, myocardial infarction, and sudden death. Public health concerns are amplified by increasing use of prescription stimulants among adults.

**Methods:**

The objective of this study was to conduct a systematic review of the evidence of an association between prescription stimulant use and adverse cardiovascular outcomes. PUBMED, MEDLINE, EMBASE and Google Scholar searches were conducted using key words related to these topics (MESH): *ADHD; Adults; Amphetamine; Amphetamines; Arrhythmias, Cardiac; Cardiovascular Diseases; Cardiovascular System; Central Nervous Stimulants; Cerebrovascular; Cohort Studies; Case–control Studies; Death; Death, Sudden, Cardiac; Dextroamphetamine; Drug Toxicity; Methamphetamine; Methylphenidate; Myocardial Infarction; Stimulant; Stroke; Safety.* Eligible studies were population-based studies of children, adolescents, or adults using prescription stimulant use as the independent variable and a hard cardiovascular outcome as the dependent variable.

**Results:**

Ten population-based observational studies which evaluated prescription stimulant use with cardiovascular outcomes were reviewed. Six out of seven studies in children and adolescents did not show an association between stimulant use and adverse cardiovascular outcomes. In contrast, two out of three studies in adults found an association.

**Conclusions:**

Findings of an association between prescription stimulant use and adverse cardiovascular outcomes are mixed. Studies of children and adolescents suggest that statistical power is limited in available study populations, and the absolute risk of an event is low. More suggestive of a safety signal, studies of adults found an increased risk for transient ischemic attack and sudden death/ventricular arrhythmia. Interpretation was limited due to differences in population, cardiovascular outcome selection/ascertainment, and methodology. Accounting for confounding and selection biases in these studies is of particular concern. Future studies should address this and other methodological issues.

## Background

There has been increasing concern that prescription stimulant use may be linked to adverse cardiovascular events such as sudden death, myocardial infarction, and stroke. Scrutiny has increased, in part, due to the burgeoning use of prescription stimulants among adults [1-3]. Older adults using prescription stimulants [4] may be particularly vulnerable to adverse cardiovascular events, given their higher background rate of cardiovascular events and comorbid conditions, higher doses of stimulants [2,5], and slower drug elimination [6].

Prescription stimulants are primarily used in the treatment of attention deficit hyperactivity disorder (ADHD), but also for obesity [7] and narcolepsy [8] as well as “off-label” indications such as depression [9], stroke rehabilitation [10], and traumatic brain injury [11]. Stimulants act by blocking reuptake of norepinephrine and dopamine as well as increasing their release into the extracellular space [12]. Stimulants may cause adverse cardiovascular events by 1) increasing blood pressure and heart rate [13-17], 2) inducing vasospasm through increased levels of circulating catecholamines [18-27], 3) causing vasculitis by inducing formation of circulating proinflammatory immunoactive gylcation end products [19,28-38], and 4) prolonging the cardiac QT interval, which is associated with torsades de pointes [39-41]. The cardiovascular epidemiological literature has shown that even modest increases in blood pressure have been associated with increased risk of adverse cardiovascular events [42-45]. Prescription stimulants have been linked to adverse cardiovascular events in case reports [24-26,35,46-49].

Safety concerns have impacted governmental regulatory policy. In 2006, the US Food and Drug Administration (FDA) issued a class-specific warning for prescription stimulants regarding potentially increased risk of adverse cardiovascular events [50]. In 2008, the American Heart Association (AHA) published a scientific statement on the use of prescription stimulants in children and adolescents [51]. It recommended obtaining a careful history and performing a physical exam prior to initiating stimulants. Reflecting the prevailing uncertainty about cardiovascular safety, the guideline was non-committal regarding the need for a pre-treatment electrocardiogram. Nor did the AHA statement specify any absolute contraindications to use of stimulants, including the presence of structural heart disease. Nor did they address prescription stimulant use in adults—the population presumably at the highest risk of adverse outcomes. Subsequently, the American Academy of Pediatrics (AAP) recommended not routinely obtaining electrocardiograms in children in a 2008 policy statement [52]. The AAP and AHA released a consensus statement later in 2008 that described obtaining an ECG prior to initiating stimulant therapy as reasonable but not mandatory, and that treatment should not be withheld on the basis of not having obtained an ECG [53]. Conversely, Health Canada recommended avoiding stimulant use in patients with symptomatic cardiovascular disease and known structural cardiac abnormalities [54]. Since then screening of children initiated on stimulants by non-cardiologists and cardiologists increased in Canada and the US [55,56]. Australian draft guidelines on ADHD completed in 2009 recommended assessment of cardiac risk factors prior to initiating stimulant use. But these guidelines remain unapproved due to concerns about the scientific integrity of some referenced studies [57].

Recently, two prescription stimulants have fallen under strict US regulatory scrutiny. Sibutramine, a compound marketed as an appetite suppressant and closely related to the amphetamine-family, was withdrawn from the US market in October 2010 by its manufacturer Abbott Laboratories, at the request of the FDA [58]. An increased risk of adverse cardiovascular events (16%) was weighed against modest weight loss. Qnexa, a combination of topiramate and the stimulant phentermine, failed to achieve FDA approval in 2010 for the treatment of weight loss due in part to concerns about cardiovascular risk [59]. This was despite its clear efficacy in weight loss. In a reversal, an FDA advisory committee voted overwhelmingly to recommend approval of the drug, persuaded in part that the benefit of treating obesity outweighs the risk of adverse events [60].

### Rationale

Only recently have observational studies begun to address whether prescription stimulants are associated with adverse cardiovascular events. Providers, patients, and policy makers need clearer guidance on the best way to balance potential benefits and harms of these rapidly increasingly used medications.

### Objective

The aim of this study was to systematically review population-based studies of children and adults that tested the association between exposure to prescription stimulants and adverse cardiovascular outcomes. Methodological challenges that face the field and suggestions for future directions of research are described.

In this review, “prescription stimulants” refer to prescribed medications in the amphetamine-family of drugs, namely comprised of amphetamine, methylphenidate, methamphetamine and their variants. Sometimes stimulants are referred to in the plural as “amphetamines” (as distinguished from the drug amphetamine which has a specific chemical structure).

## Methods

### Methods

For the systematic review**,** studies were considered using the following criteria: 1) retrospective or prospective population-based study, 2) children or adults as participants, 3) prescription stimulant use as the independent variable, and 4) one or more hard cardiovascular outcomes as the dependent variable and primary outcome. Blood pressure, pulse, and EKG changes—established physiological effects of stimulants—were not considered hard clinical events. PUBMED, MEDLINE, EMBASE, and Google Scholar databases were searched for studies published in English in peer-reviewed journals between January 1, 1990 and April 1, 2012 using MESH terms and keywords: ADHD; Adults; Amphetamine; Amphetamines; Arrhythmias, Cardiac; Cardiovascular Diseases; Cardiovascular System; Central Nervous Stimulants; Cerebrovascular; Cohort Studies; Case–control Studies; Death; Death, Sudden, Cardiac; Dextroamphetamine; Drug Toxicity; Methamphetamine; Methylphenidate; Myocardial Infarction; Stimulant; Stroke; Safety. In addition, we hand-searched potentially relevant studies cited in the reference section of electronically identified articles. Titles and abstracts were screened for inclusion/exclusion, and full text versions were retrieved. Selected population-based studies of children were categorized separately from studies of adults for the purpose of comparisons. Studies were assessed for bias at both the study and outcome levels. Catchment, comparison groups, exposure and outcome ascertainment, statistical power and methodologies were assessed.

## Results

### Population-based observational studies of prescription stimulant use

**Figure 1:**
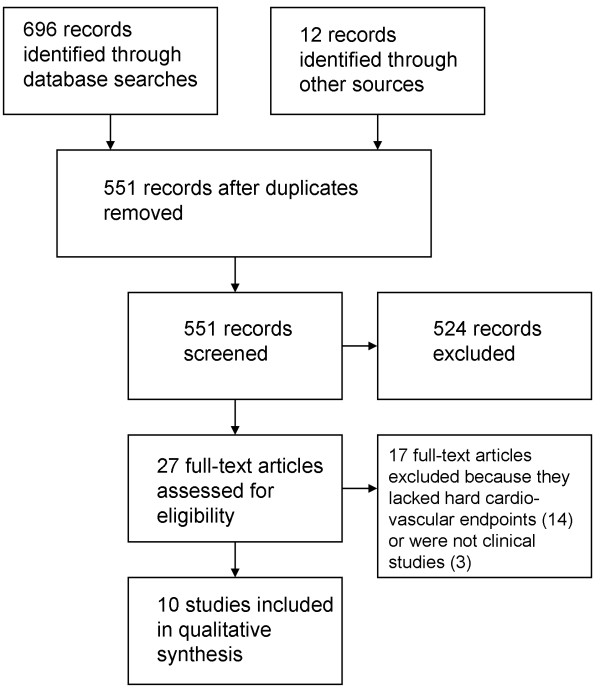
Systematic Review Flowchart

Overall, 551 unique records were identified in searches. Most records were excluded based on review of titles and abstracts due to not being topical to prescription stimulants and not having appropriate endpoints. Twenty-seven full-text articles were assessed for eligibility. Most (14) were eliminated due to lack of hard cardiovascular endpoints. Ten studies met the inclusion criteria and were included in the qualitative synthesis (Figure 1). These studies, using large population-based datasets specifically designed to detect a signal of cardiovascular harm associated with medical use of stimulants, found mixed results (Table 1).

**Table 1:** Population-Based Observational Studies of Prescription Stimulants and Adverse Cardiovascular Events

**Year**	**Author**	**Study-Type/Drugs**	**Data Source**	**Population**	**Independent Variable**	**Outcome Variables**	**Statistical Approach**	**Result/Conclusion**
2007	Winterstein et al.	Retrospective cohort/methylphenidate, amphetamines, and pemoline	Florida Medicaid	3 to 20 years old with new diagnosis of ADHD (124,932 person-years)	Current use, former use, nonuse	1) Cardiac Death	Cox regression	No increased risk of cardiac death or hospitalizations. Observed 20% increase in hazard for CV ER visits with current use over nonuse.
						2) First Cardiovascular (CV) Hospitalization		
						3) First CV ER visit		
2009	Winterstein et al.	Retrospective cohort/methylphenidate, amphetamine salts	Florida Medicaid	3 to 20 years old with diagnosis of ADHD (52,783 person-years) and newly started on methylphenidate or amphetamine salts	Current use, former use	First CV ER visit	Cox regression	No difference in risk of first CV ER visit between methylphenidate and amphetamine salts
2009	Holick et al.	Retrospective matched cohort/atemoextine, “ADHD medication” (methylphenidate, amphetamine salts)	Health insurance database (Ingenix Research DataMart)	18 years or older, atomoxetine initiators (n = 21,606) matched to “ADHD medication” initiators (n = 21,606)	Current use, recent use, past use, nonuse	1) Cerebrovascular accident (CVA)	Propensity scoring, Cox regression	No increased risk of CVA or TIA with atomoxetine compared to stimulants. Increased risk of TIA with ADHD medication compared to general population (hazard ratio 3.44, 95% confidence interval 1.13-10.60).
						2) Transient ischemic attack (TIA)		
2009	McCarthy et al.	Descriptive cohort/methylphenidate, dextroamphetamine, atomoxetine	UK General Practice Research Database	2 to 21 years old (18,637 person-years)	Ever used	Sudden death	Incident rate ratio, standardized mortality ratio	No increased risk of sudden death with stimulant and atomoxetine use.
2009	Gould et al.	Matched case–control/amphetamine, dextroampheamine, methamphamine, methylphenidate	State vital statistics offices	7 to 19 years old, sudden death associated with stimulant use (n = 926) versus matched controls (n = 564; motor vehicle accident fatalities)	Stimulant use immediately prior to death	Sudden death	Logistic regression analysis of matched pairs	Increased risk of sudden death associated with stimulant use (odds ratio 7.4, 95% confidence interval 1.4 to 74.9).
2011	Schelleman et al.	Retrospective matched cohort/amphetamines, atomoxetine, methylphenidate	Medicaid (5 states) and health insurance database (HealthCore)	3 to 17 years old incident ADHD medication users matched to nonusers	Current use	1) Sudden death/ventricular arrhythmia	Proportional hazards regression	No statistically significant difference in rates of outcomes between exposed and non-exposed.
						2) Stroke		
						3) Myocardial infarction (MI)		
						4) Composite stroke/MI		
2011	Cooper et al.	Retrospective matched cohort/amphetamines, methylphenidate, pemoline, atomoxetine	Medicaid (Tennessee and Washington), health insurance databases (Kaiser Permanente California and OptumInsight Epidemiology), state death certificates, National Death Index	2 to 24 years old ADHD medication users matched to nonusers (2,579,104 person-years)	Current use, former use, nonuse	1) Sudden cardiac death	Cox regression, propensity scoring	No increased risk of serious cardiovascular events for current users (adjusted hazard ratio 0.75, 95% confidence interval 0.31 to 1.85).
						2) Stroke		
						3) Myocardial infarction		
2011	Habel et al. (Journal of the American Medical Association)	Retrospective matched cohort/amphetamines, methyldphenidate, pemoline, atomoxetine	Tennessee Medicaid, health insurance databases (Kaiser Permanente California and OptumInsight Epidemiology), HMO Research Network, state death records, National Death Index	Adults 25 to 64 years old ADHD medication users matched to nonusers (806,182 person-years)	Current use, indeterminate use, former use, remote use, nonuse	1) Myocardial infarction (MI)	Poisson regression adjusted by cardiovascular risk score and confounders; propensity scoring and external adjustment methods used in secondary analyses	No increased risk of serious cardiovascular events (MI/SCD/stroke) for current users compared to nonusers (adjusted rate ratio 0.83, 95% confidence interval 0.72-0.96)
						2) Sudden cardiac death (SCD)		
						3) Stroke		
2012	Schelleman et al.	Retrospective matched cohort/methylphenidate	Medicaid (5 states) and health insurance database (HealthCore)	18 years and older methylphenidate users (n = 43,999) matched to nonusers (n = 175,955)	Current use, nonuse	1) Sudden death/ventricular arrhythmia	Proportional hazards regression adjusted for age and data source; propensity scoring as secondary analysis	Increased risk of sudden death or ventricular arrhythmia for current users (adjusted hazard ratio 1.84, 95% confidence interval 1.33 to 2.55). No increased risk of stroke, myocardial infarction, or composite stroke/MI.
						2) Stroke		
						3) Myocardial infarction (MI)		
						4) Composite stroke/MI		
2012	Olfson et al.	Retrospective cohort/methylphenidate, amphetamines	Health insurance database (MarketScan Research Databases)	6 to 21 years old with ADHD and non know cardiovascular risk factors, treated with stimulants (n = 89,031) or not treated with stimulants (n = 82,095)	Current use, former use, nonuse	1) Severe cardiovascular events (AMI, subarachnoid hemorrhage, stroke, ischemic heart disease, sudden death, respiratory arrest	Logistic regression adjusted for age, days from index diagnosis, and propensity score	No analysis for severe cardiovascular events was performed due to only one incident event in the entire cohort. For less severe cardiovascular events, there was no increased risk associated with stimulant use compared to nonuse (adjusted odds ratio 0.69, 95% CI 0.42-1.12)
						2) Less severe cardiovascular events (angina pectoris, cardiac dysrhythmias, transient cerebral ischemia)		

### Children and adolescents

In Winterstein et al.’s study of 55,383 Florida Medicaid beneficiaries, 3 to 20 years old, no increase in cardiac death or hospitalizations was observed [61]. However, there was a 20% increased risk in cardiac-related emergency room visits among current users of stimulants. A second study by Winterstein et al., compared the risk between methylphenidate and amphetamine salt medication preparations [62]. In 2,131,953 Florida Medicaid beneficiaries, 3 to 20 years old, no difference was found in risk of emergency room visits for cardiac reasons between the two medication groups.

McCarthy et al. studied a UK database of patients, 2 to 21 years old, who were prescribed methylphenidate, dexamphetamine, or atomoxetine to determine whether use was associated with a greater risk of sudden death [63]. In 18,637 patient-years, six of the seven deaths were determined to not be cases of sudden death, and one death was of indeterminate cause. Compared to a reference rate of sudden death, the investigators did not find a significantly increased risk of sudden death associated with use of these medications.

Gould et al. conducted a case–control study of 564 cases of sudden death among youth 7 to 19 years old, matched with 564 deceased passenger victims in motor vehicle accidents. Stimulant use (amphetamine, dextroamphetamine, methamphetamine, methylphenidate) was associated with increased odds of sudden death (odds ratio = 7.4; 95% CI 1.4 to 74.9) [64].

Schelleman et al. (2011) studied youth 3 to 17 years (n = 241,417), derived from a 5-state Medicaid database and a 14-state health insurance database [65]. Hazard ratios for incident exposure to stimulants (amphetamines, atomoxetine, methylphenidate, or combination therapy) compared to non-exposure were not significantly elevated for primary outcomes of sudden death/ventricular arrhythmia, stroke, myocardial infarction, and composite stroke/myocardial infarction. Cardiovascular outcomes were validated via medical records and independent adjudication. However, only 48% of requested records were obtained, and the adjudicators were not reported as blinded. In secondary analyses using claims-based outcomes (not validated) and prevalent (i.e. not incident) stimulant use, significant associations were found between sudden death/ventricular arrhythmia and methylphenidate, atomoxetine, and any ADHD medication.

Recently in 2011, Cooper et al. conducted a retrospective matched cohort study of children and young adults 2 to 24 years (n = 1,200,438) using Medicaid (Tennessee and Washington), health insurance, state death certificates, and the National Death Index [66]. Current use of ADHD medications was not significantly associated with increased risk for serious cardiovascular events (sudden death, stroke, and myocardial infarction). Additional secondary analyses, with adjustments to inclusion criteria and independent and dependent variables did not find a significant relationship between ADHD medication use and cardiovascular events. Analyses were adjusted for baseline and time-varying covariates as well as site-specific propensity scores. The study benefitted from review of medical records (79% availability) to perform end-point validation [67].

Olfson et al. in 2012 studied 6 to 21 year olds (n = 171,126) with ADHD and no known cardiovascular risk factors, comparing stimulant users to nonusers [68]. The investigators intended to evaluate the risk of “severe cardiovascular events” which included sudden death, stroke, acute myocardial infarction, and respiratory arrest. But only one incident event in the entire cohort was discovered and no analysis was conducted due to lack of power. The risk of “less severe cardiovascular events” among persons with ADHD—comprising angina pectoris, cardiac dysrhythmias, and transient cerebral ischemia—was not significantly different between stimulant users and nonusers.

### Adults

Holick et al.’s population-based study of adults 18 years and older (adults ≥ 65 years old were included) compared use of atomoxetine (a non-stimulant used in the treatment of ADHD that is associated with increases in blood pressure [69]; n = 21,606) to use of stimulant ADHD medications (n = 21,606) [70]. Propensity scoring was used to match atomoxetine and prescription stimulant users. Use of atomoxetine was not associated with a either a greater risk of stroke or transient ischemic attack (TIA), compared to stimulant ADHD medications. In a secondary analysis, atomoxetine and stimulant users, matched to a general population cohort (n = 42,993), had a significantly increased risk of TIA, but not stroke.

The study by Habel et al. represents the largest and most comprehensive study of ADHD medications and cardiovascular outcomes in adults to date (n = 443,198) [71]. In this retrospective cohort study of adults 25 through 64 years old, each ADHD medication user was matched to two nonusers. Medical charts, autopsy reports and death certificates were obtained and adjudicated for myocardial infarction (MI), sudden cardiac death (SCD), and stroke outcomes where available. Adjustment for confounding included the use of a cardiovascular risk score (CRS), which summarized cardiovascular risk factors. Unexpectedly, current use of ADHD medications compared to nonuse was significantly protective against serious cardiovascular events (MI, SCD, or stroke; adjusted rate ratio 0.83, 95% CI, 0.72-0.96). The authors submitted a more detailed report to the AHRQ, using only MI and SCD as outcomes, with the same conclusion that the results did not support an association between ADHD medication use and the risk of MI and SCD [72].

The most recent study of adults (18 years and older), by Schelleman et al. (2012), matched methylphenidate users (n = 43,999) to nonusers (n = 175,955) and found an increased risk of sudden death or ventricular arrhythmia among users (adjusted hazard ratio 1.84, 95% CI 1.33 to 2.55) [73]. No statistically significant difference in risk was found for stroke, myocardial infarction, and a combined endpoint of stroke/myocardial infarction. In a secondary analysis, the risk of all-cause death was significantly increased for methylphenidate users compared to nonusers (adjusted hazard ratio 2.38, 95% CI 2.20 to 2.56).

## Discussion

Six out of seven studies in children and adolescents did not show an increased risk of adverse cardiovascular events (Table 1). Because the incidence of cardiovascular events in children is low, the power needed to detect an association between prescription stimulants and events is extraordinarily high, leading to an increased likelihood of a false negative outcome (type II error). The study among the seven with the most power, by Cooper et al. (n = 1,200,438), could not rule out a doubling of risk due to the low incidence of serious cardiovascular events (n = 81) in those 2 to 24 years old. Lack of statistical power was an issue in other studies. Winterstein et al. stated that 16 times more person-years would have been required for their study to detect a doubling of the hazard ratio [61]. The Schelleman et al. study (2011) found no validated cases of ADHD-associated stroke and myocardial infarction, strongly suggesting that the study was under-powered. Likewise Olfson et al. found no severe cardiovascular events in the study cohort. The study by McCarthy et al. was also underpowered. There were other limitations in the studies of children and adolescents. The study by Gould et al., the only one to find an association between prescription stimulants and adverse cardiovascular outcomes in children and adolescents, was unable match on race and geographical region, both of which may have confounded the association. Additionally, exposure to prescription stimulants was not derived from pharmacy data, but rather from informants, medical records, toxicology findings, and death certificates. Misclassification of exposure was also a concern, if persons who were illicitly using street methamphetamine were classified as exposed to prescription stimulants. In Schelleman et al.’s study (2011), analyses were not adjusted for confounders, nor selection bias.

The three studies of adults had mixed findings, with two of the studies showing a safety signal regarding prescription stimulant use and cardiovascular outcomes. Holick et al. found an increased risk of transient ischemic attack (TIA), but not stroke, among ADHD medication initiators compared to the general population in a secondary analysis. However, unlike the primary analysis, propensity scoring was not used to match the general population cohort with the combined atomoxetine and prescription stimulant use cohorts. A strength of this study was that investigators attempted to corroborate drug exposure and stroke/TIA outcomes in claims data with medical records. In the Schelleman et al. study (2012), methylphenidate use was associated with a 1.8-fold increased risk of sudden death or ventricular arrhythmia, but was not associated with increased risk of stroke, myocardial infarction or combined stroke/myocardial infarction. However, primary analyses were adjusted for only age and data source. In post hoc analyses to account for confounding, propensity scores were used and found similar results to the primary analyses, with attenuated, but still significantly increased risks of sudden death/ventricular arrhythmia and all-cause death. The largest and most ambitious of the three studies of adults, by Habel et al., did not find an increased risk of MI, sudden cardiac death, and stroke among adults with short median exposure (median 0.33 years) to ADHD medications. In fact, statistical results suggested that ADHD medications were protective against serious cardiovascular events [71] which the authors acknowledged as “biologically implausible” [72]. With survey data from Kaiser Permanente, external adjustment methods were used to account for unmeasured confounders among the entire cohort—including the Tennessee Medicaid population which was sicker and used ADHD medications for shorter periods of time on average [72]. ADHD medication users were more educated and less likely to be black or Hispanic, leading the investigators to state that the true estimate of the adjusted risk ratio was likely higher but was masked due to healthy user bias. In general the study population had a low rate of cardiovascular risk factors. A strength of this study included the numerous sensitivity analyses that were performed, including that of new users, current users versus remote users, and the construction of a propensity score using variables included in the cardiovascular risk score. These analyses did not suggest a significant association between prescription stimulant use and adverse cardiovascular outcomes. Although the authors had 80% power to detect a rate ratio of 1.23 for the primary analysis (current use versus nonuse), they concluded that “a modestly elevated risk cannot be ruled out, given limited power and a lack of complete information on some potentially important risk factors and other factors related to use of these medications.” The study could not make any conclusions about the elderly, as patients 65 years or older were not analyzed.

### Considerations for future studies: Methodological challenges and proposed strategies

In the following section, we summarize the main methodological challenges and outline considerations and strategies for future studies. **Low Absolute Rate of Cardiovascular Events.** Randomized clinical trials (RCTs) are not feasible because of the low incidence rate of stroke, acute myocardial infarction and cardiovascular death, the need for very large numbers of patients, and ethical concerns. Due to power considerations, future work will need to rely on very large population-based cohorts. **Hard Clinical Outcomes**. Future investigations should be designed with hard clinical endpoints such as death, myocardial infarction, and stroke as the primary outcomes of interest. While prior literature has demonstrated that small increases in blood pressure lead to increased cardiovascular morbidity and mortality on a population-basis [45], there is no proven association between stimulant-induced soft endpoints (e.g. increases in blood pressure and heart rate) and hard cardiovascular endpoints. **Study Populations**. Because the baseline rates of sudden death, stroke, and myocardial infarction in children are so low, increases in risk may not have a large absolute impact. However in adults a modest increase in risk could have significant clinical impact. Thus an important strategy is to use an enriched population, such as older adults, as greater than 80% of deaths due to heart disease occur in those 65 years or older [74]. **Measurement of Prescription Stimulant Use**. The relationship of time and dose of stimulants to the risk of adverse cardiovascular events is unknown. It may be that there are both acute and chronic risks with prescription stimulant use. It stands to reason that a higher dose of stimulants incurs more risk than a lower dose, but this has not been proven [27]. Sensitivity analyses, where modeling of use is varied, can help address this problem. **Confounders/Selection Bias/Control Groups.** Accounting for confounding and selection bias is the greatest challenge for observational studies seeking to study stimulants and the risk of cardiovascular events. **Confounding**. Observational studies attempt to account for confounding by including known confounders as covariates in a multivariate analysis. Of particular concern is confounding by indication, when a variable is present in the non-exposed, but also an indication for the exposure of interest (e.g. treatment of obesity with prescription stimulants). Confounding by *contraindication*, where a patient avoids treatment due a contraindication, is also a potential problem. One way to address confounding by indication or contraindication is to limit the study population, by excluding persons with the problematic indication(s). But this comes at the expense of generalizability. **Selection Bias**. Persons that use prescription amphetamines differ in important ways from persons that are not prescribed amphetamines. Some of these ways are known and measured, and can normally be adjusted for in an analysis, such as comorbid medical risk factors for cardiovascular events. But groups differ in ways that are unmeasured. These unmeasured factors are known in some cases (e.g. poor diet), and unknown in others. Propensity scoring and instrumental variable techniques are two more sophisticated statistical approaches used to adjust for biases in observational data. To date, five studies have used propensity scoring [66,68,70,71,73]. Studies that do not account for selection bias are extremely difficult to interpret, as it becomes unclear whether the risk is due to the independent variable (prescription stimulant use), or the differences in the populations that are compared. Even with studies that do use propensity scoring, such as the study by Habel et al., it can be difficult to determine whether such techniques adequately accounted for unmeasured confounders. For example, inability of these techniques to fully control for healthy user effects probably explains why Habel et al. found a lower risk-adjusted rate of adverse cardiovascular events among stimulants users. Use of propensity scoring and instrument variables offers the promise of adjusting for selection biases. As the ability to control for confounding and selection bias increases, so does the confidence in the results of such studies.

## Conclusions

Seven of the ten studies included in this systematic review did not find an association between prescription stimulant use and adverse cardiovascular outcomes. Six of the seven studies of children and adolescents did not find an association between prescription stimulant use and adverse cardiovascular outcomes. Low incidence of adverse cardiovascular outcomes among children and adolescents in the general population hampered these studies. In adults, however, a safety signal—prescription stimulant use associated with adverse cardiovascular outcomes—was demonstrated in two of three studies. Of primary concern in these studies were problems of confounding and selection bias. Future studies must, in particular, address these issue. Studies of at-risk populations, including the elderly and those with a high burden of cardiovascular disease are needed as well.

## Competing interests

AW has consulted as an expert witness for a private university. EH has no competing interests.

## Authors’ contributions

AW and EH both contributed to the design and conception, drafting and revision of the manuscript, and have read and approved the final manuscript.

## Pre-publication history

The pre-publication history for this paper can be accessed here:

http://www.biomedcentral.com/1471-2261/12/41/prepub
